# Comparative effects of dairy, hybrid and plant-based protein blends (including fibre fortification) on amino acid profiles and gut microbiota adaptations: The Promephy study

**DOI:** 10.1016/j.crfs.2026.101359

**Published:** 2026-02-19

**Authors:** Justin Roberts, Joseph Lillis, Jeff Mercer, Helen Mercer, Ioannis Kostopoulos, Lotte Dopheide, Ashley G.B. Willmott, Sebastian Tims, Matthew Furber, Ardy Van Helvoort, Jorge Marques Pinto

**Affiliations:** a Faculty of Science & Engineering, Anglia Ruskin University, Cambridge, CB1 1PT, UK; b Danone Research & Innovation, Utrecht, 3584 CT, The Netherlands; c Faculty of Health, Medicine and Social Care, Anglia Ruskin University, Cambridge, CB1 2LZ, UK; d Cambridge University Hospitals NHS Foundation Trust, Cambridge, CB2 0QQ, UK; e Institute of Nutrition and Translational Research in Metabolism (NUTRIM), Maastricht University, The Netherlands

**Keywords:** Protein, Plant-based, Amino acids, Uremic toxins, Microbiota, Fibre

## Abstract

This study investigated the acute and sustained effects of different protein interventions on postprandial amino acid profiles, uremic toxins, faecal fermentation metabolites and microbiota composition in healthy volunteers. Sixteen participants (7 males, 9 females; mean [SD]: 37 [9] years; 69.4 [10.2] kg; 1.74 [0.08] m) completed four 2-week intervention periods, separated by a 6-week wash-out. For each intervention, participants were allocated 50 g d^−1^ protein from either a milk protein isolate (MPI), a hybrid blend (HYB), a plant-based blend (PB) or a PB blend with added mixed fibres (PBF) in addition to habitual dietary intake. Pre and post intervention, participants underwent laboratory assessment of amino acid profiling (4 h post consumption of 20 g protein from the allocated product), along with stool sample collection for microbiota sequencing.

Plasma essential amino acid (EAA) profiles and incremental area under the curve (iAUC) were lower for both PB products at baseline compared with MPI and HYB (MPI: 89,397 [18,025]; HYB: 87,301 [16,920]; PB: 65,350 [17,111]; PBF: 66,595[20,827] μmol·240 min L^−1^, *p* < 0.001). Following intervention, EAA profiles decreased further with both PB products, remaining lower than both MPI and HYB (MPI: 83,038 [20,983]; HYB: 76,341 [16,741]; PB: 56,075 [17,045]; PBF: 56,170 [15,213] μmol·240 min L^−1^, *p* < 0.001). After the 2-week intervention, L-leucine iAUC was lower for all products compared with MPI (MPI: 19,219 [4444]; HYB: 16,776 [3291]; PB: 12,003 [3008]; PBF: 11,948 [2991] μmol·240 min L^−1^, *p* ≤ 0.016), highlighting the importance of fortification or increased protein intake with PB containing products. PB inclusion into products resulted in minor increases in indoxyl sulfate within-intervention (*p* ≤ 0.039) which may be potentially beneficial in lower concentrations. Both HYB and PBF interventions resulted in increased saccharolytic metabolite production in relation to proteolytic metabolites (*p* ≤ 0.008). PBF resulted in a significant reduction in Peptostreptoccaceae compared to all products (*p* ≤ 0.007), as well as an increase in Marinifilacaeae (*p* = 0.014), which may support gastrointestinal health. The inclusion of fibre into protein interventions may therefore be beneficial for modulating metabolic ‘fates’ of protein, as well as supporting relative taxonomic changes which may regulate gastrointestinal integrity and function.

## Highlights

• It is unclear if plant-protein and fibre impacts amino acid uptake or gut microbiota.
• Essential amino acid uptake was lower with plant-protein than diary or hybrid blends.
• Hybrid and fibre-fortified plant-protein enhanced saccharolytic activity by week 2.
• Only plant-protein with fibre impacted Peptostreptoccaceae and Marinifilacaeae taxa.

## Graphical abstract

**Figure:**
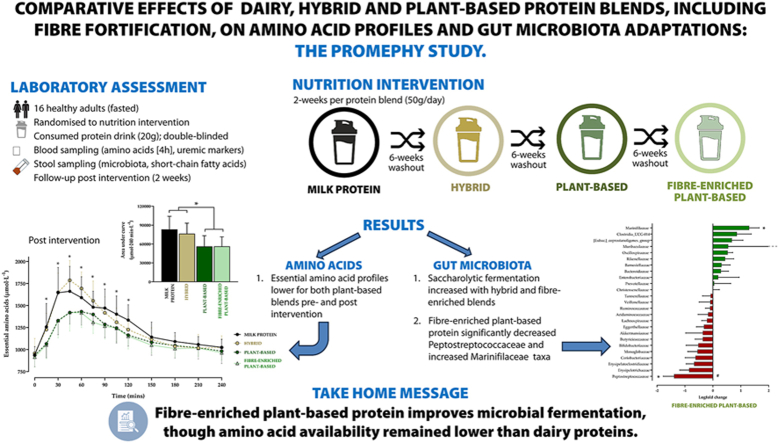

## 1 Introduction

Protein, particularly from high quality sources such as eggs, milk, poultry, beef or soy, is an essential dietary component for human function (Wolfe et al., 2024), providing amino acids that support adaptive remodelling of muscle and connective tissue, through to hormone production and immune modulation (Li et al., 2007; Phillips, 2014). The rate and quantity of amino acid uptake, particularly the indispensable (essential) amino acids especially L-leucine, have been positively associated with fractional synthetic rate of muscle (Boirie et al., 1997; Groen et al., 2015; Katsanos et al., 2006; Moore et al., 2009; Witard et al., 2014), which may be critically important in disease or acute care conditions (De Waele et al., 2021), as well as beneficial during post-exercise recovery (Tang et al., 2009). Whilst protein digestion/absorption kinetics are influenced by caloric content (Luiking et al., 2016), the specific type of protein can also be impactful. Whey protein, as example, has been shown to result in faster uptake, and greater total systemic amino acid levels when compared with casein, hydrolysed casein, soy, rice or pea protein (Liu et al., 2019; Pennings et al., 2011; Rogers et al., 2024). Plant-based proteins, containing limiting amino acids, are considered lower quality (Pinckaers et al., 2021), and even when consumed in combination (to achieve a more complete amino acid profile) may still have lower bioavailability dynamics compared to dairy protein (Ajomiwe et al., 2024; de Marco Castro et al., 2022). Despite known health benefits, other components within plant-based structures (e.g. anti-nutritional factors such as trypsin and chymotrypsin inhibitors, tannins, phytic acid and lectins) may impact amino acid uptake, rendering plant-based alternatives inferior to animal based proteins (Ajomiwe et al., 2024). This presents challenges for those opting for a flexitarian or solely plant-based diet, and coupled with current sustainability and healthy living agendas, highlights an increased demand for alternative protein solutions, such as protein isolate blends and/or fortification with other nutrients such as specific amino acids or fibre (Berrazaga et al., 2019).

Another consideration is the amount of protein required per day. Whilst recommended daily intakes in Europe reside at 0.8 g· kg^−1^·d^−1^, such intakes may be inadequate in relation to certain medical conditions, acute care, ageing or regular physical activity status (Deer and Volpi, 2015). Protein intakes >1.2 g· kg^−1^·d^−1^ may be required to support the needs of such populations, and >1.6 g· kg^−1^·d^−1^ for those undertaking strenuous exercise (Phillips et al., 2016; Stokes et al., 2018; Witard et al., 2025). Typically, only a small percentage of proteins reach the colon, depending on protein source and amount consumed (Yao et al., 2016). However, with increased intakes, the question arises whether higher amounts of protein remain undigested and reach the colon, leading to proteolytic fermentation by resident microbiota and altered microbiome composition.

The microbiota typically favours saccharolytic fermentation of fibres (indigestible carbohydrates; Canfora et al., 2019; Korpela, 2018), resulting in short-chain fatty acid (SCFA) production by beneficial microbes, alongside wider systemic health benefits (Blaak et al., 2020; Boven et al., 2025; Korpela, 2018). However, lower fibre intake associated with a Western diet (Barber et al., 2020), as well as higher protein intakes, can result in a microbiota ‘switch’ to proteolytic fermentation (Aguirre et al., 2016; Bartlett and Kleiner, 2022; Canfora et al., 2019; Rodriguez-Romero et al., 2022), predominantly in the distal colon (Canfora et al., 2019; Peled and Livney, 2021; Windey et al., 2012). Increased proteolytic fermentation may facilitate gene expression of gram-negative bacteria, increased production of uremic toxins, ammonia and undesired fermentation metabolites, including branched-chain fatty acids (BCFAs). These can ultimately lead to negative health consequences as a result of epithelial damage and permeability, immune cell activation and pro-inflammatory responses (Jackson et al., 2024). The balance between saccharolytic and proteolytic fermentation may therefore be of importance for host physiology (Jardon et al., 2022). It is also unclear whether differences exist in the ‘metabolic fates’ between animal and plant-based protein sources in terms of microbiota changes, and the balance between proteolytic/saccharolytic fermentation (Blachier et al., 2019).

Despite lower ‘bioavailability’ profiles, other compounds found in plant-based sources (e.g. fibre, micronutrients) may have other important metabolic benefits and may potentially offset the negative effects of high protein dairy intakes (e.g. proteolytic fermentation, bloating). As most plant-based protein isolates and concentrates (e.g. pea and soy) contain relatively low amounts of fibre, provision of additional, and preferably diverse fibres, could modulate microbiota composition favouring saccharolytic fermentation (Boven et al., 2025; Jackson et al., 2024). Whether sustained use of plant-based or fibre-enriched strategies could impact protein ‘bioavailability’ is also currently unknown, particularly when protein isolates are used.

The aim of this study was therefore to assess the acute and sustained effects of different protein blends on postprandial amino acid profiles, uremic toxins, faecal fermentation metabolites and microbiota composition in healthy volunteers. In comparison to a high-quality protein source (milk protein isolate), evaluation of plant-based isolates (soy and pea), fibre-enriched plant-based isolates, and a hybrid (whey, casein, pea, soy) blend was undertaken. It was hypothesized that amino acid profiles would be greater with milk protein and hybrid blends, but that profiles would be improved with plant-based isolate blends after sustained higher protein intake, with a particular emphasis on the essential amino acids including L-leucine. It was also hypothesized that sustained intake of high levels of protein from plant-based blends, especially when enriched with diverse fibres, would lead to enhanced saccharolytic fermentation compared to milk protein isolate.

## 2 Materials and methods

### 2.1 Study objectives, ethical approval and clinical trial registration

The main objectives of this study were: i) to compare postprandial serum amino acids profiles (including total and, in particular, essential amino acids, especially L-leucine) before and after two weeks of daily consumption of milk protein isolate (MPI), a hybrid blend of dairy and plant protein isolates (HYB; casein, whey, pea, soy), a plant protein isolate blend (PB; pea, soy), or a PB blend with added mixed fibres (PBF); and ii) to compare changes in taxonomic microbiota composition after two weeks of daily consumption of the above four protein interventions.

This study was undertaken following institutional ethical approval from the Faculty of Science and Engineering Research Ethics Panel, Anglia Ruskin University (Ethical approval no. ETH2223-3364) and was registered with ClinicalTrials.gov (ID: NCT05669612). All laboratory-based procedures were conducted in accordance with the Declaration of Helsinki (World Medical Association, 2025) at the Human Physiology Laboratory, Anglia Ruskin University, Cambridge, UK.

### 2.2 Power calculation and participant recruitment

Initial *a priori* power assessment was undertaken utilising data from similar research (Liu et al., 2019). Based on the objective of assessing the incremental area under the curve (iAUC) particularly for the sum of the essential amino acids (EAAs), it was estimated that a minimum of 9 participants would be required to observe a significant difference in the change from baseline in iAUC of EAA between a plant-based (pea-soy) protein mix and a dairy-based (whey-casein) protein mix based on repeated measures. Sample size calculation was based on the following assumptions: a log-normal distribution for the iAUC of EAAs; an intra-subject correlation of 0.5; a 1-β of 80% (power); and a mean difference on the log-scale of 0.29. Due to the longitudinal nature of this study, a drop-out rate of 40% was expected, therefore it was estimated that a target sample size of 15 participants would be required for the study.

In order to be considered for this clinical trial, participants were required to be: aged between 18 and 50 years with a body mass index (BMI) between 18.5 and 24.9 kg·m^−2^, have the ability to provide informed consent, and demonstrate willingness and ability to comply with all study requirements, including dietary adherence and, where applicable for female participants, willingness to use a method of birth control during study participation. Participants were excluded from this study if they had: experienced any significant medical or surgical event in the three months prior to starting the study; used antibiotics or medications affecting gastrointestinal function within the preceding three months; or participated in another clinical trial within the previous month. Further exclusion criteria included: smoking; an average alcohol intake >14 units per week for females or >21 units per week for males; engagement in high-volume or high-intensity exercise routines (>20h per week, e.g. trained elite athletes); if participants followed strict or restrictive diets (e.g., vegan, ketogenic, paleo or weight loss) or had consumed any nutritional or protein supplements within six weeks of starting the main study; had food allergies or intolerances relevant to the intervention (such as pea, soy, corn, gluten, lactose); were pregnant or lactating; or had a known history or current diagnosis of: cancer, gastrointestinal, cardiovascular, respiratory, bleeding, renal, hepatic, thyroid, immune, or metabolic disorders, including diabetes mellitus. Participants were also excluded if blood haemoglobin levels were considered low at pre-screening (<12.09 g·dL^−1^ [<7.5 mmol· L^−1^] for males and <11.28 g· dL^−1^ [<7.0 mmol· L^−1^] for females).

A total of 24 participants volunteered for the study and provided written informed consent; however, 7 participants were not included based on the main inclusion/exclusion criteria during pre-screening. Therefore, 17 participants began the main study. One participant was withdrawn from the study at trial 2 due to protocol deviation. The remaining 16 participants satisfactorily completed the full study protocol, with study power deemed sufficient based on *a priori* assessment. General characteristics of the 16 participants are displayed in Table 1.

**Table 1:** Participant characteristics.

Characteristics	Total *n* = 16
Age (years)	37 (9)
Gender	Males: *n* = 7; Females: *n* = 9
Country of origin^a^	United Kingdom [12]; Poland [1]; Greece [1]; China [1]; Caribbean [1]
Body height (m)	1.74 (0.08)
Body mass (kg)	69.4 (10.2)
Body mass index (kg∙m^−2^)	22.9 (1.6)
Haemoglobin (Hb; g∙dL^−1^)	14.1 (0.9)

Data mean (SD). Hb reported as part of pre-trial assessment.

a All participants were based in the United Kingdom.

### 2.3 Study protocol and laboratory procedures

**Fig. 1:**
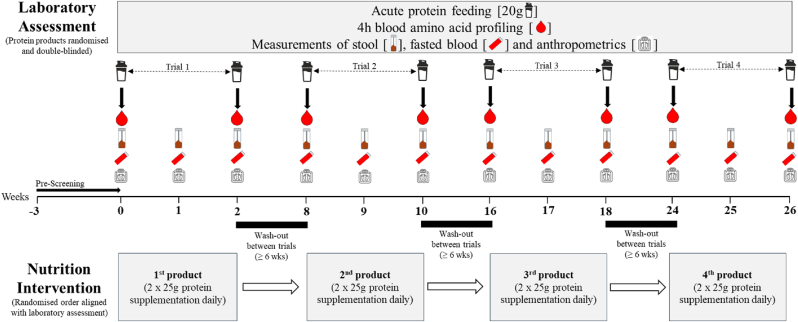
Schematic overview of the full study protocol including laboratory-based assessment and at home nutritional interventions.

This study employed a longitudinal, randomised, controlled, cross-over, repeated measures design, with participants completing four 2-week intervention periods (nutrition interventions 1-4), separated by at least a 6-week wash-out period between interventions. The full study protocol is shown in Fig. 1.

Following the pre-screening phase and confirmation of eligibility, participants were randomised into one of four nutrition intervention groups using a computer-generated counter-balanced approach (randomiser.org) to ensure appropriate sequence generation (order of interventions) and balanced distribution of nutrition products within each intervention. All products were prepared independently of the research team, and supplied in sealed, opaque, label-coded containers by Danone Research & Innovation, Utrecht, The Netherlands, ensuring a double-blinded approach.

*Laboratory Assessments (Trials 1-4):* All laboratory assessments were conducted under standardised conditions, within the Human Physiology Laboratory, Anglia Ruskin University, UK using the same protocol. Each 2-week trial period consisted of three laboratory visits, involving the following:

Visit 1 (Baseline amino acid profile assessment) - During the three days prior to each visit 1, participants completed a weighed food diary based on habitual dietary intake, collected an at-home stool sample (2 x 13 mL tubes immediately frozen locally; Boettger GmbH & Co., Germany) and completed an associated Bristol Stool Chart (on the same day of the stool sample collection). The Bristol Stool Chart assesses stool consistency based on subjective evaluation using representative images (scaled 1-7) accompanied with written description of stool form types (Lewis and Heaton, 1997). Stool samples were immediately frozen at −80 °C upon receipt until analysis.

Participants limited exercise in the 24 h prior, consumed a standardised evening meal (based on estimated maintenance calories targeting ∼ 10 kcal· kg^−1^, of which 50% carbohydrate, 20% protein, 30% fat, as described elsewhere (Roberts et al., 2021)), and arrived at the laboratory in a fasted (≥10h) and euhydrated state. Upon arrival, body mass (Seca digital scales, Hamburg, Germany) and height (Seca stadiometer, Hamburg, Germany, at initial visit only) were recorded, followed by the collection of a resting venous blood sample into duplicate 4 mL serum clot activator Vacuette™ tubes (Greiner Bio-One GmbH, Kremsmunster, Austria) via cannulation, which remained in situ for necessary repeated sampling. This sample constituted time (T) = −20 min, with a repeated resting sample collected at T = −5 min. Following this, participants were then provided with one of four randomly assigned protein drinks (20 g vanilla-flavoured protein mixed with 200 mL water, with an additional 50 mL rinse to ensure complete delivery), which was consumed within 10 min. Further blood samples were collected at 15, 30, 45, 60, 75, 90, 105, 120, 150, 180, 210 and 240 min after study product intake as part of a 4h amino acid profile assessment. All blood samples were assessed for amino acids (see blood marker analysis), with the additional assessment of uremic markers from the first resting sample. Wholeblood samples were centrifuged for 10 min at 2000×*g*, with aliquoted serum samples immediately frozen at −80 °C until analysis.

Throughout the session, participants completed symptom and gastrointestinal tolerance questionnaires at predetermined intervals (before product intake, at 90 and 240 min). Covered tolerance domains were nausea, vomiting, abdominal distension, burping, flatulence, dry mouth and thirst. Water intake was standardised with 200 mL provided at 95 and 185 min only. At the conclusion of visit 1, participants received a two-week supply of their allocated protein supplement and were instructed to consume 2 × 25 g protein servings daily in addition to habitual dietary intake. Compliance was tracked using a supplement log and verified with home weighing scales.

Visit 2 (Mid-trial check in) - After one week, participants returned to the laboratory for a mid-trial check in having adhered to the same standardised pre-evening meal and fasting protocol. For this session, body mass was recorded on arrival, along with collection of a second stool sample (2 x 13 mL) and accompanying Bristol Stool Chart. Following confirmation of adherence to the study protocol, a resting venous blood sample (2 x 4 mL) was collected for assessment of mid-trial changes in uremic markers.

Visit 3 (Follow-up amino acid profile assessment) - At the end of the two-week supplementation period, participants repeated all procedures conducted during visit 1 to evaluate changes in amino acid profiles. Following visit 3, participants entered a 6-week washout period before commencing the same study protocol with an alternate protein formula.

### 2.4 Dietary and exercise activity monitoring

Over the course of each 2-week trial period, habitual dietary and hydration intake were recorded via a mobile based application (MyFitnessPal, Inc., San Francisco, CA, USA) for the 3 days leading into each trial, in addition to 3 randomly selected days, including one weekend day. Participants were provided with a personal login and guidance instructions to support detailed dietary tracking. Dietary tracking was checked by a member of the research team at regular intervals for consistency and assessed using Nutritics Professional Dietary Analysis software (Nutritics Ltd., Co. Dublin, Ireland) by the same researcher.

In addition to dietary assessment, participants were required to complete a standardised daily exercise activity diary for the 3 days prior to first laboratory visit for each trial and across the fourteen consecutive days supplementation period. Importantly, during this time, participants were instructed to maintain habitual diet and exercise practises to ensure consistency across all four trials. Session type, mean session heart rate, exercise duration and session rating of perceived exertion ([sRPE] using a standard 0–10 visual analogue scale) were recorded following the completion of each training session as reported elsewhere (Foster et al., 2001; Roberts et al., 2021).

### 2.5 Nutrition interventions

Participants were randomly allocated 4 different protein formulas across the full study. The nutritional and amino acid composition of all 4 products is shown in Table 2. For one of the plant-based formulas, an additional 3 g of mixed fibres (per 25 g protein) was included. The mixed fibres specifically consisted of arabinoxylan, oat beta-glucan, pectin and resistant starch in a ∼5:2:2:1 ratio. These types were selected to be representative of the major fibre types obtained from fibre food sources in the diet such as grains, cereals, fruits, legumes and vegetables (Ionita-Mindrican et al., 2022; Lovegrove et al., 2017; Stephen et al., 2017); and were carefully selected to cover a variety of physiochemical and chemical fibre properties including soluble, insoluble, and various backbone structures (Ionita-Mindrican et al., 2022; Stephen et al., 2017). To improve the taste of the products, sucralose and vanilla flavours were added.

**Table 2:** Nutritional composition of the protein products (A), and independent amino acid analysis (B).

(A)		Milk protein isolate (MPI)	Hybrid combination (HYB)	Plant-based combination (PB)	Plant-based with mixed fibres (PBF)
Formula composition	Whey (%)	20	36	0	0
	Casein (%)	80	24	0	0
	Soy (%)	0	20	67	67
	Pea (%)	0	20	33	33
Product serving for laboratory trials (g)		25.8	24.3	24.4	28.5
Protein quantity per serve (g)		20.0	20.0	20.0	20.0
Fibre content per serve (g)		0.0	0.4	0.9	3.5

(B)	Independent amino acid composition∗ Values in grams per 20g protein			
	MPI	HYB	PB	PBF
L-Alanine	0.7	1.0	0.9	0.9
L-Arginine	0.7	1.0	1.6	1.6
L-Aspartic Acid	1.7	2.4	2.3	2.4
L-Cysteine	0.2	0.3	0.2	0.2
L-Glutamic Acid	4.7	4.3	3.8	4.0
L-Glycine	0.4	0.6	0.8	0.9
L-Proline	2.1	1.5	1.0	1.0
L-Tyrosine	1.1	0.8	0.8	0.7
L-Serine	1.2	1.2	1.1	1.1
L-Histidine	0.6	0.5	0.5	0.5
L-Isoleucine	1.1	1.2	0.9	1.0
L-Leucine	2.1	2.1	1.8	1.7
L-Lysine	1.8	1.8	1.3	1.3
L-Methionine	0.6	0.4	0.2	0.2
L-Phenylalanine	1.1	1.0	1.1	1.1
L-Threonine	1.0	1.2	0.8	0.8
L-Tryptophan	0.3	0.3	0.3	0.3
L-Valine	1.3	1.2	1.0	1.0
				
Σ EAA	9.8	9.6	7.9	7.9
Σ NEAA	13.4	13.4	13.0	13.5
Σ BCAA	4.6	4.4	3.7	3.6
Σ AA	22.7	22.5	20.4	20.8

N = nitrogen; EAA = Essential amino acids; NEAA = Non-essential amino acids; BCAA = Branched-chain amino acids. ∗Specific amino acid analysis undertaken independently of product evaluation of protein quantity, explaining higher levels of Total EAAs and AAs for MPI and HYB.

For the main laboratory postprandial amino acid assessment, participants received 20 g protein (mixed with 200 mL water, and an additional 50 mL rinse) from each of the allocated nutrition products with required volume of powdered mix determined from independent certification (Vitablend Nederland BV, The Netherlands). Across each 2-week nutrition intervention period, participants consumed 50 g additional protein of the same allocated product daily in two divided doses (25 g in the morning, and 25 g in the evening away from food). Each 25 g protein dose was mixed with 300 mL water, including an additional 50 mL rinse to ensure complete delivery. For PBF, the additional fortification equated to an extra 6 g fibre provided per day.

### 2.6 Blood marker analysis

*Serum amino acids:* All analysis was undertaken at Danone Research & Innovation, Utrecht, The Netherlands. Serum blood levels of 22 amino acids (alanine, arginine, aspartic acid, asparagine, citrulline, cysteine, glutamic acid, glutamine, glycine, histidine, isoleucine, leucine, lysine, methionine, ornithine, phenylalanine, serine, taurine, threonine, tryptophan, tyrosine, valine) were analysed as previously described (Henderson and Brooks, 2010; Terrlink et al., 1994). All chemicals and reagents (perchloric acid, L-norvaline, o-phthaldialdehyde [OPA], 3-mercaptopropionic acid, sodium phosphate dibasic dihydrate, sodium borate, sodium azide, diluted hydrochloric acid, methanol and acetonitrile) were purchased from Sigma-Aldrich (Merck Life Science N.V., The Netherlands) and of analytical grade unless stated otherwise. Briefly, after precipitation of proteins and polypeptides with perchloric acid, samples were centrifuged for 10 min at 1400×*g*. The internal standard L-norvaline was added to the resulting supernatant followed by derivatisation with OPA in the presence of 3-mercaptopropionic acid. In total 1.0 μL of the OPA-derivatives was injected onto an ACQUITY Premier BEH C18 column (1.7 μm particles, 100 × 2.1 mm) connected to an ultra-fast liquid chromatography (UFLC) system (Nexera 40 Series, Shimadzu, Japan) using fluorimetry as detection at excitation wavelength of 340 nm and emission at 455 nm. Eluent A was prepared by weighing 1.75 g sodium phosphate dibasic dihydrate, 3.80 g sodium borate and 0.25 g sodium azide, 1000 g demineralised water and 2.5 mL diluted hydrochloric acid in demineralised water. For eluent B, 176.9 g methanol, 178.0 g acetonitrile and 50.0 g water were used. Samples were degassed and filtered over a 0.45 μm filter. Calibration curves were generated for each amino acid, including use of an internal standard (QC) from which quantification was undertaken. Positive iAUC was assessed using the trapezoidal rule and confirmed using a Time Series Response Analyser (TSRA) tool (Narang et al., 2020).

*Serum uremic markers:* Assessment of serum uremic markers (hippuric acid, kynurenic acid, kynurenine, indoxyl sulfate, p-cresyl glucuronide and p-cresyl sulfate) was undertaken at the Department of Pharmaceutical Sciences, University of Utrecht, The Netherlands using LC-MS/MS as previously described (Ahmed et al., 2025). For chemicals and reagents, LC-MS grade chemicals were purchased for uremic markers from Sigma-Aldrich (St. Louis, Missouri, USA), along with acetic acid (for solvent A). Separately, p-cresyl glucuronide and p-cresyl sulfate were purchased from AlsaChim (Illkirch-Graffenstaden, France). Acetonitrile (HPLC-S grade, for Solvent B), water (LC-MS grade) and methanol (HPLC grade) were purchased from Biosolve (Valkenswaard, The Netherlands). Analytical grade formic acid was obtained from Merck (Darmstadt, Germany). A Milli-Q® Advantage A10 Water Purification System (Merck, The Netherlands) was used to produce ultrapure water. For isotope-labelled internal standards: 13C6-indoxyl sulfate and d5-hippuric acid were purchased from Cambridge Isotope Laboratories (Andover, MA, USA); d4-kynurenine and d5-kynurenic acid were purchased from CDN Isotopes (Pointe-Claire, Quebec, Canada); d7-p-cresyl sulfate (potassium salt) was purchased from IsoSciences (Ambler, PA, USA) and d7-p-cresyl glucuronide was purchased from Toronto Research Chemicals (North York, Ontario, Canada).

An Accela LC system coupled to a TSQ Quantum Ultra triple quadrupole mass spectrometer with heated electrospray ionisation (Thermo Fisher Scientific, San Jose, CA, USA) was used for uremic marker analysis. Xcalibur software (version 2.07, Thermo Fisher Scientific, San Jose, CA, USA) was used for controlling, data recording and processing. Chromatographic separation was undertaken using a Waters ACQUITY UPLC HSS T3 column (100 mm × 2.1 mm, 1.8 μm particle size) equipped with an ACQUITY UPLC HSS T3 VanGuard pre-column (5 mm × 2.1 mm, 1.8 μm particle size), maintained at 40 °C. Analytes were separated using a mixed isocratic–gradient elution at a flow rate of 0.45 mL·min^−1^. Solvent A (0.2% [v/v] acetic acid) and solvent B (acetonitrile) were used in the mobile phase. Initially, 5% B was held for 1 min, before being linearly increased to 15% B over 1 min, and then to 20% B for 1 min. To flush the column, solvent B was then increased linearly to 80% within 1 min. Re-equilibration was performed by returning to 5% B and holding for 2 min.

Samples were pretreated prior to LC-MS/MS analysis, being diluted twofold with ultrapure water with 20 μL diluted serum aliquoted into 1.5 mL Eppendorf tubes. Following this, 30 μL of acetonitrile (4 °C) with isotope-labelled internal standards was added. Samples were vortex-mixed for 5 min and centrifuged at 4000×*g* for 5 min. From the supernatant, a 35 μL sample was aliquoted to a 1 mL round-bottom well of a polypropylene 96-deep well plate and further diluted with 200 μL of ultrapure water. The plate was gently shaken before being placed in the autosampler for analysis. Ultrapure water was used as a surrogate matrix for preparing calibration curves and quality control samples. Calibration curves were constructed via linear regression using 8 concentrations of the standard for each analyte. Peak areas were normalised to their respective labelled internal standards, with reliability determined via four quality-control samples. Following this, concentrations of the uremic markers were subsequently determined.

### 2.7 Stool sample analysis

*Fermentation metabolite analysis in stool samples:* All analysis was undertaken at Danone Research & Innovation, Utrecht, The Netherlands. All chemicals and reagents (phosphate-buffered saline [PBS], formic acid, 2-ethylbutyric acid, and trichloroacetic acid [TCA]) were purchased from Sigma-Aldrich (Merck Life Science N.V., The Netherlands) and of analytical grade unless stated otherwise. Stool samples were diluted 1:10 in ice-cold PBS and homogenised by adding glass beads (3 mm in diameter) followed by vigorous shaking for 5 min at maximum speed (Heidolph™ Multi Reax, Heidolph Scientific Products GmbH, Schwabach, Germany). The resulting supernatant was transferred to a new tube and centrifuged for 3 min, 15,000×*g* at 4 °C. The cleared supernatant was diluted 14 x in 5% formic acid including the internal standard 2-ethylbutyric acid. The SCFAs (propionic, acetic, n-butyric and n-valeric acids), BCFAs (iso-butyric and isovaleric acids) and internal standard were quantified by means of gas chromatography equipped with flame ionisation detection (Shimadzu-GC2050, Kyoto, Japan) and hydrogen as mobile phase. SCFA and BCFA concentrations were calculated via a calibration curve and the internal standard 2-ethylbutyric acid.

For the determination of lactate, supernatants were centrifuged for 10 min at 19,000×*g* at 4 °C, followed by an enzyme inactivation step by incubating the sample for 10 min at 100 °C and subsequently cleared by centrifugation for 10 min at 19,000×*g*. D-and L-lactate were determined enzymatically using an Enzytec™ kit (R-Biopharm AG, Darmstadt, Germany) according to the manufacturer instructions. The Ammonia Assay Kit (rapid) (K-AMIAR, Megazyme, Ireland) was used to quantify ammonia levels. Prior to following the instructions provided with the kit, samples were fixated with TCA (30% w/v) to a final concentration of 3% TCA per sample. Stool pH was determined by applying a glass microelectrode pH meter (Mettler Toledo SevenMulti™, Ohio, USA) equipped with glass electrode (Mettler Toledo InLab® Micro, Ohio, USA) directly into the stool sample.

*Faecal DNA extraction*: For DNA extraction of the stool samples the previously described extraction method using a QIAmp PowerFecal Pro DNA Kit (Qiagen, Hilden, Germany) combined with additional bead beating steps was performed (Schipper et al., 2024), except that 0.2 g of stool sample was used.

*Microbiota analysis*: All analysis was undertaken at Danone Research & Innovation, Singapore. For chemicals and reagents, the internal control (PhiX) was purchased from Illumina (California, USA). A mock microbial community DNA standard (ZymoBIOMICS D6306) as positive control was purchased from Zymo Research (California, USA); whereas nuclease-free water as negative control was purchased from Thermo Scientific (Vilnius, Lithuania). A two-step PCR workflow was performed for library preparation according to the Illumina 16S sample preparation guide (Illumina, California, USA), as described previously (Schipper et al., 2024). An internal control of 5% PhiX, a positive control (mock microbial community DNA standard) and a negative control (nuclease-free water) were included in every plate that was processed. Pooled libraries were sequenced on the Illumina Miseq platform using a MiSeq v3 reagent cartridge (600 cycles) for paired-end sequencing (read lengths of 2 x 300 bp) to obtain a minimum of 50,000 raw Paired-End sequences per sample.

### 2.8 Statistical analysis

All main statistical analyses were conducted using IBM SPSS Statistics (version 28, IBM Corp., Armonk, NY, USA). Data were assessed for normality using Shapiro-Wilk tests and visual inspection of Q-Q plots, with outliers identified by inspection of box plots >1.5 interquartile range. Where necessary, variables were log-transformed to meet model assumptions. A mixed linear model (MLM) approach was employed to assess differences in amino acid profile, uremic markers and fermentation metabolites, accounting for repeated measures and intra-subject variability. For amino acid analysis fixed effects included product, trial and timepoint, with participant specified as a random effect. iAUC, maximum concentration (Cmax), time to maximum concentration (Tmax), as well as uremic markers and fermentation metabolite parameters were analysed using MLMs with product and visit as fixed effects, and participant as a random effect. To compare relative changes in iAUC, Cmax and Tmax, the model included product as a fixed effect and participant as a random effect. A one-way ANOVA was employed to assess for any differences in either dietary intake or exercise patterns relative to each product. Post-hoc comparisons were adjusted using Bonferroni correction. Results are reported as mean (SD), with a significance threshold set at p < 0.05.

*Microbiota analysis:* Alpha diversity, including Shannon's diversity index and Chao1 richness index, were calculated using the vegan R package (version 2.6-4). Differences were assessed within and between groups using a linear mixed model (timepoint, donor) using lmer R package (version 1.1.37). Beta diversity was performed using non-metric multidimensional scaling (NMDS) based on Bray-Curtis distances and visualised using the plot_ordination function from the phyloseq R package (McMurdie and Holmes, 2013). Permutational multivariate analysis of variance (PERMANOVA)/Adonis2 tests were conducted using the vegan Package in R (version 2.6-4) on the Bray-Curtis distances. Additionally, the Bray-Curtis distances between the timepoints within each product were calculated using the vegan R package (version 2.6-4) and the differences of dissimilarity values between the timepoints were evaluated by pairwise comparisons via the Dunn test (dunn.test R package version 1.3.6) using *p* adjusted values. Further analysis of the community composition and structure was undertaken using the microbiome R package (version 1.30.0). Differential abundance analyses were performed with DESeq2 (version 1.48.0) on the 16S rRNA sequencing data to account for the abundance variation for each donor and at different time points. Spearman's correlation coefficient was used for analyses of correlations between gut microbial abundances (logfold change) and iAUC for TAAs, EAAs and L-leucine (logfold change). Results are reported as mean (SD) unless stated, with a significance threshold set at p < 0.05. All figures were produced using Graphpad Prism (version 10.2.2).

## 3 Results

### 3.1 Compliance, dietary and physical activity assessment

All participants completed the study with exemplary protocol compliance (>99% adherence across interventions). No habitual dietary differences in energy intake, macronutrient content or fibre intake were observed between the four product periods (*p* > 0.05; Table 3). Additionally, habitual physical activity parameters, such as frequency, duration and training load were also comparable across conditions (*p* > 0.05; Supplementary Table 1).

**Table 3:** Dietary assessment (not including additional supplementation) relative to each intervention, including product adherence.

		MPI	HYB	PB	PBF
EI	kcal·d ^−1^	2109.6 (536.9)	2077.9 (532.3)	1948.3 (452.0)	1940.9 (475.6)

PRO	g·d^−1^	90.2 (25.0)	92.9 (20.2)	86.5 (22.7)	91.5 (26.3)
	g·kg^−1^	1.3 (0.4)	1.4 (0.3)	1.3 (0.4)	1.3 (0.3)
	% of EI	17.2 (3.0)	18.2 (3.6)	18.2 (5.2)	18.9 (4.0)
CHO	g·d^−1^	233.0 (69.4)	214.2 (76.1)	218.8 (75.7)	214.1 (58.4)
	g·kg^−1^	3.3 (0.6)	3.1 (0.9)	3.1 (0.8)	3.1 (0.8)
	% of EI	44.2 (5.3)	40.7 (8.7)	44.5 (7.6)	44.6 (9.2)
Fibre	g·d^−1^	26.2 (10.8)	26.2 (14.3)	24.6 (8.1)	23.2 (11.1)

Fat	g·d^−1^	87.8 (23.9)	91.3 (22.9)	79.4 (19.7)	76.8 (31.2)
	g·kg^−1^	1.3 (0.3)	1.3 (0.4)	1.1 (0.2)	1.1 (0.4)
	% of EI	37.6 (5.2)	40.0 (7.5)	36.6 (4.9)	35.2 (8.0)
Product adherence	%	99.8 (0.8)	99.8 (0.9)	100.0 (0.0)	100.0 (0.0)

EI = Energy intake; PRO = Protein; CHO = Carbohydrate. No significant differences observed.

### 3.2 Blood amino acid profiles

**Fig. 2:**
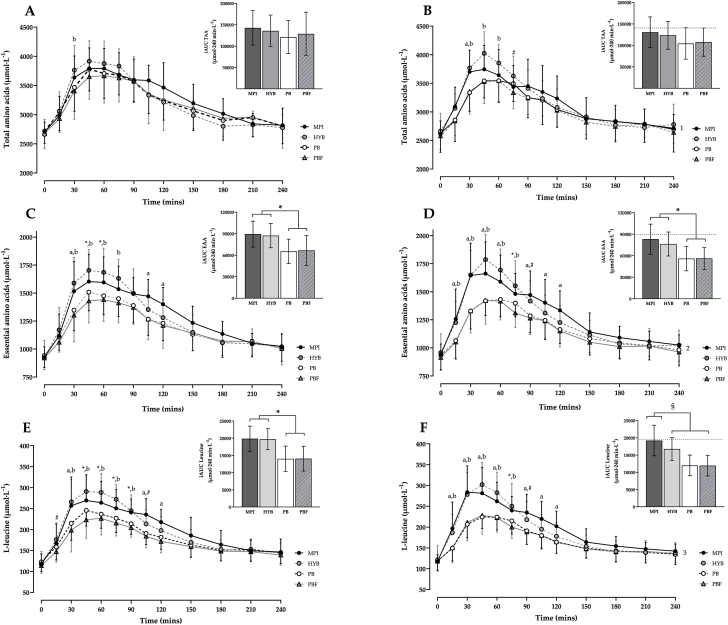
Plasma amino acid profiles following consumption of 20 g protein from MPI, HYB, PB or PBF at baseline [A, C, E] and end of trial period [B, D, F] for total amino acids (TAA; [A, B]), essential amino acids (EAA; [C, D]) and L-leucine (E, F). Inserts demonstrate iAUC with dotted line highlighting mean peak for MPI from baseline. For main graphs, ^*a*^ denotes MPI was significantly different to PB and PBF (*p* ≤ 0.01); ^*b*^ denotes HYB was significantly different to PB and PBF (*p* ≤ 0.039); ∗ denotes MPI was significantly different to PBF (*p* ≤ 0.012); ^#^ denotes HYB was significantly different to PBF (*p* ≤ 0.045). ^1^ highlights significant difference for MPI, PB and PBF compared to baseline (*p* < 0.001). ^2^ highlights significant difference for PB and PBF compared to baseline (*p* ≤ 0.002). ^3^ highlights significant difference for HYB and PB compared to baseline (*p* ≤ 0.01). For inserts, ∗ = significant difference for PB and PBF compared to both MPI and HYB (*p* < 0.001); ^§^ = significantly different to MPI (*p* ≤ 0.016).

*Total amino acids:* For TAAs (Fig. 2A and B; Table 4), a significant interaction effect was observed between product and trials (F = 3.28; *p* = 0.02), and within-trial over time (F = 2.35; *p* < 0.001). At baseline, TAA concentrations were higher at 30 min in HYB than in PB (*p* = 0.039) and PBF (*p* = 0.006). By week 2, further pronounced differences were observed in that TAAs were greater at 30 min for both MPI and HYB compared with both PB products (*p* ≤ 0.01); and remained significantly higher for HYB compared to both PB and PBF at 60 min (*p* ≤ 0.025), and to PBF only at 75 min (*p* = 0.045). Within-product analysis demonstrated significant reductions in TAAs pre-post intervention for all products (*p* < 0.05), except HYB. No significant differences were observed for TAA iAUC, Cmax, Tmax or relative changes within or between trials for any of the products (*p* > 0.05).

**Table 4:** Mean (SD) and delta (Δ) amino acid data for TAAs, EAAs and L-leucine (LEU) relative to each product intervention.

		MPI			HYB			PB			PBF		
		Baseline	Week2	Δ	Baseline	Week2	Δ	Baseline	Week2	Δ	Baseline	Week2	Δ
TAAs	iAUC	142,620 (40,267)	130,527 (35,886)	−12093 (42,677)	135,674 (37,059)	123,672 (32,478)	−12002 (46,821)	121,094 (38,569)	104,452 (36,645)	−16642 (50,895)	128,436 (50,998)	107,632 (32,844)	−20803 (58,480)
	Cmax	4112 (292)	3910 (449)	−201 (433)	4065 (373)	4064 (333)	−1 (440)	3914 (399)	3670 (307)	−244 (360)	3936 (350)	3646 (356)	−291 (403)
	Tmax	66 (28)	56 (30)	−9 (39)	57 (20)	47 (9)	−11 (24)	56 (19)	58 (22)	3 (34)	65 (24)	52 (15)	−13 (29)
EAAs	iAUC	89,397 (18,025)	83,038 (20,983)	−6359 (17,374)	87,301 (16,920)	76,341 (16,741)	−10960 (17,260)	65,350∗ (17,111)	56,075∗ (17,045)	−9275 (19,306)	66,595∗ (20,837)	56,170∗ (15,213)	−10426 (22,323)
	Cmax	1757 (187)	1761 (293)	4 (226)	1768 (209)	1808 (216)	41 (189)	1568∗ (170)	1482∗ (124)	−86 (151)	1553∗ (153)	1470∗ (186)	−84 (171)
	Tmax	59 (25)	55 (23)	−4 (33)	57 (18)	46 (9)	−12 (21)	57 (16)	64 (20)	7 (22)	65 (22)	53 (14)	−12 (27)
LEU	iAUC	19,870 (3664)	19,219 (4444)	−651 (3190)	19,702 (3048)	16,776^#^ (3291)	−2926 (2217)	14,012∗ (3712)	12,003^#^ (3008)	−2009 (2928)	14,052∗ (3596)	11,948^#^ (2991)	−2104 (3552)
	Cmax	304 (40)	307 (61)	3 (57)	304 (45)	308 (53)	4 (36)	256∗ (36)	234∗ (28)	−22 (28)	246∗ (34)	236∗ (37)	−10 (35)
	Tmax	54 (21)	48 (21)	−6 (29)	56 (18)	43 (9)	−12 (21)	56 (16)	60 (17)	5 (18)	51 (20)	50 (14)	−1 (25)

iAUC = incremental area under the curve (μmol·240 min L^−1^); Cmax = maximum concentration (μmol·L^−1^); Tmax = time to reach Cmax (min). ∗ = significantly different to both MPI and HYB relative to corresponding week (*p* ≤ 0.005). ^#^ significantly different to MPI at week 2 (*p* ≤ 0.016).

*Essential amino acids:* For EAAs (Fig. 2C and D; Table 4), a significant interaction effect was observed between product and trials (F = 2.87; *p* = 0.035), and within-trial over time (F = 5.60; *p* < 0.001). During baseline assessment, EAA concentrations were greater at 30 min in MPI and HYB compared with both PB products (*p* ≤ 0.005). Between 45 and 60 min, HYB exhibited significantly higher EAAs than PB and PBF (*p* < 0.001), whereas EAAs were higher for MPI compared with PBF only at the same time points (*p* ≤ 0.012). By 75 min, EAA concentrations were only greater with HYB compared with both PB products (*p* ≤ 0.002); however, between 105 and 120mins, a ‘cross-over’ was observed with only MPI demonstrating higher EAAs compared to both PB products (*p* ≤ 0.004). By week 2, a faster uptake of EAAs was observed at 15 min for MPI and HYB compared with both PB products and remained significantly greater until 60 min (*p* ≤ 0.009). At 75 min, EAAs remained higher for HYB compared with PB and PBF (*p* ≤ 0.012), whereas concentrations for MPI were higher than PBF only (*p* = 0.004). Between 90 and 120 min, a similar ‘cross-over’ effect was observed for MPI with higher EAA concentrations than PB and PBF (*p* ≤ 0.004), whereas values were only higher for HYB compared with PBF at 90 min (*p* = 0.015). Within-product analysis demonstrated significant reductions in EAAs pre to post intervention for both PB products (*p* < 0.05), whereas MPI and HYB remained unchanged (*p* > 0.05).

When assessing overall iAUC, no interaction effects were observed between trials, however a within-trial effect was demonstrated (F = 14.44; *p* < 0.001). At both baseline and week 2, iAUC was significantly lower for both PB products compared with MPI and HYB (*p* < 0.001). In a similar manner, a within-trial effect was found for Cmax (F = 29.06; *p* < 0.001; Table 4), with both MPI and HYB eliciting higher peak EAA concentrations compared to both PB products at baseline and week 2 (*p* < 0.001). Fibre fortification did not impact EAA absorption, with PBF showing similar reductions in iAUC and Cmax to PB. Tmax was not significantly altered between trials for any of the products.

*L-leucine:* For L-leucine, a significant interaction effect was observed between product and trials (F = 2.70; *p* = 0.044), and within-trial over time (F = 6.63; *p* < 0.001) (Fig. 2E and F; Table 4). At baseline, HYB resulted in a rapid uptake of L-leucine by 15 min compared with PBF (*p* = 0.04), which remained significantly higher than both PB products until 90 min (*p* ≤ 0.021), and PBF only at 105 min (*p* = 0.033). L-leucine concentration was also higher for MPI compared to both PB products at 30, and 105-120 min only (*p* ≤ 0.004), and additionally compared to PBF at 45-90 min (*p* ≤ 0.006). By week 2, an increased uptake of L-leucine was observed for both MPI and HYB compared to both PB products particularly at 15 min, remaining greater through to 60 min (*p* ≤ 0.003). The L-leucine profile continued to remain elevated between 90 and 120 min for MPI compared with both PB products (*p* ≤ 0.003; Fig. 2F). Within-product analysis demonstrated significant reductions in the L-leucine profile over the intervention for both HYB and PB products compared to baseline (*p* ≤ 0.01).

When assessing iAUC for L-leucine, no interaction effects were observed between product and trials, however a within-trial effect was demonstrated (F = 23.56; *p* < 0.001). At baseline, MPI and HYB elicited a significantly higher iAUC compared with PB and PBF (*p* < 0.001), with no differences observed between MPI and HYB or between PB and PBF (*p* = 1.000). However, at week 2, iAUC was lower for all products compared to MPI (*p* ≤ 0.016). In a similar manner, a within-trial effect was found for Cmax (F = 39.86; *p* < 0.001; Table 4), with both MPI and HYB eliciting higher peak L-leucine concentrations compared to both PB products at baseline and week 2 (*p* ≤ 0.005). Tmax was not significantly altered between trials for any of the products (*p* > 0.05).

### 3.3 Uremic metabolites

Uremic metabolites exhibited several product-specific alterations over the intervention period (Table 5). Indoxyl sulfate (F = 8.27; *p* < 0.001) increased significantly from baseline to week 1 in the HYB, PB, and PBF groups (*p* ≤ 0.035) and additionally to week 2 for HYB and PB (*p* ≤ 0.039). In contrast, p-cresyl glucuronide (F = 6.30; *p* = 0.002) only increased significantly in the PBF group at week 2 compared with baseline (*p* = 0.017). For p-cresyl sulfate (F = 4.62; *p* = 0.004), HYB demonstrated significantly increased concentrations from baseline to week 1 (*p* = 0.004), followed by a significant reduction from week 1 to week 2 (*p* = 0.024). No significant changes in hippuric acid, kynurenic acid or kynurenine were observed across the intervention for any of the products (*p* > 0.05).

**Table 5:** Mean (SD) uremic markers relative to each product intervention.

	MPI			HYB			PB			PBF		
	Baseline	Week1	Week2	Baseline	Week1	Week2	Baseline	Week1	Week2	Baseline	Week1	Week2
Hippuric acid	6.83 (4.44)	4.89 (3.47)	5.70 (3.70)	5.90 (3.60)	5.42 (2.76)	6.19 (3.35)	5.71 (3.19)	6.02 (3.24)	4.32 (2.47)	6.15 (4.23)	6.53 (3.91)	6.00 (2.66)
Indoxyl sulfate	3.79 (2.46)	4.72 (2.20)	4.59 (1.79)	4.42 (1.83)	5.87∗ (2.05)	5.85∗ (1.91)	4.44 (2.07)	6.66∗ (2.29)	6.01∗ (2.33)	4.88 (1.82)	6.81∗ (2.99)	5.88 (2.20)
Kynurenic acid	0.04 (0.01)	0.04 (0.01)	0.04 (0.01)	0.04 (0.01)	0.05 (0.02)	0.05 (0.01)	0.04 (0.01)	0.05 (0.01)	0.04 (0.01)	0.04 (0.01)	0.05 (0.01)	0.04 (0.01)
Kynurenine	1.90 (0.66)	1.94 (0.50)	1.82 (0.35)	1.96 (0.57)	2.09 (0.54)	1.90 (0.42)	1.81 (0.60)	2.01 (0.56)	1.95 (0.67)	1.95 (0.43)	1.92 (0.47)	1.85 (0.27)
p-cresyl glucuronide	0.06 (0.05)	0.10 (0.08)	0.07 (0.06)	0.08 (0.09)	0.11 (0.07)	0.10 (0.08)	0.08 (0.08)	0.11 (0.07)	0.08 (0.05)	0.07 (0.06)	0.12 (0.10)	0.13∗ (0.08)
p-cresyl sulfate	47.23 (32.70)	57.39 (31.09)	57.00 (29.50)	54.48 (35.94)	80.03 # (43.23)	58.85 (27.66)	48.96 (32.77)	60.58 (29.67)	61.16 (34.60)	60.51 (32.61)	69.70 (31.90)	78.11 (37.28)

Units in μM. ∗ = significantly different to baseline within condition only (*p* ≤ 0.039); ^#^ significantly different to baseline and week 2 within condition (*p* ≤ 0.024).

### 3.4 Fermentation metabolites

For fermentation metabolites (Table 6), an interaction effect was observed between product and visit for acetic acid (F = 2.31; *p* = 0.037), with PB eliciting higher concentrations compared to MPI at week 2 (mean difference: +14.25 mmol·kg^−1^; *p* = 0.01). Within-product analysis also indicated that only HYB resulted in a significant increase in acetic acid and n-butyric acid (F = 2.89; *p* = 0.037) from week 1 to week 2 (*p* < 0.02). No other significant differences were observed, including for ammonia or pH (*p* > 0.05; Table 6). When variables were collated (Table 7), a significant interaction effect was observed for ΣSCFAs, specifically increasing by + 20.66 mmol·kg^−1^ from week 1 to 2 for HYB only (*p* = 0.013). Between product differences were observed between PB and HYB at week 1 (*p* = 0.046), and between MPI and PB at week 2 (*p* = 0.007). As a proxy measure of saccharolytic: proteolytic fermentation, the ratio between ΣSCFAs: ΣBCFAs + ammonia highlighted a within-intervention effect (F = 5.96, *p* = 0.003), with a significant increase from week 1 to 2 for both HYB and PBF (*p* ≤ 0.008). No other significant differences were observed.

**Table 6:** Mean (SD) fermentation metabolites, including total lactate, ammonia and pH relative to each product intervention.

	MPI			HYB			PB			PBF		
	Baseline	Week1	Week2	Baseline	Week1	Week2	Baseline	Week1	Week2	Baseline	Week1	Week2
Acetic acid	38.94 (14.12)	37.29 (20.18)	32.92 (16.11)	36.40 (15.71)	31.27 (14.06)	43.67∗ (22.66)	37.75 (16.32)	41.59 (25.58)	47.17 # (19.69)	42.97 (17.93)	36.97 (19.77)	37.78 (17.92)
Propionic acid	11.12 (3.89)	12.22 (6.79)	11.08 (4.92)	11.18 (4.88)	10.31 (6.08)	13.44 (6.31)	12.56 (5.48)	14.36 (10.10)	15.05 (6.61)	11.45 (3.73)	11.25 (5.01)	12.06 (5.72)
n-butyric acid	10.14 (4.66)	9.21 (4.49)	8.74 (6.71)	10.21 (5.86)	8.23 (3.69)	13.05∗ (8.39)	10.84 (6.42)	12.61 (10.45)	13.49 (8.27)	11.69 (7.78)	11.05 (6.18)	9.56 (4.78)
n-valeric acid	1.56 (0.49)	1.98 (1.07)	1.56 (0.61)	1.81 (0.89)	1.46 (0.64)	1.79 (0.80)	2.01 (1.03)	2.13 (1.33)	2.23 (1.04)	1.62 (0.77)	1.71 (0.87)	1.39 (0.52)
Iso-Butyric acid	1.66 (0.56)	2.05 (0.96)	1.83 (0.79)	1.95 (1.26)	1.64 (0.84)	1.66 (0.67)	2.14 (0.95)	2.19 (1.35)	2.28 (1.20)	1.68 (0.68)	2.03 (1.02)	1.56 (0.68)
Iso-Valeric acid	2.24 (0.79)	2.80 (1.56)	2.47 (1.28)	2.56 (1.99)	2.19 (1.36)	2.21 (0.98)	2.84 (1.58)	3.03 (2.08)	3.20 (1.87)	2.30 (1.03)	2.93 (1.46)	2.08 (1.09)
Total lactate	5.33 (2.83)	4.38 (1.99)	3.95 (2.03)	5.67 (4.44)	6.51 (3.40)	7.40 (3.68)	4.53 (2.96)	4.90 (2.69)	3.22 (1.66)	3.95 (2.95)	5.07 (4.24)	6.36 (3.51)
Ammonia	24.06 (13.39)	26.76 (11.40)	23.30 (13.11)	27.15 (16.18)	26.59 (9.42)	24.68 (10.86)	28.54 (14.10)	25.93 (7.82)	27.93 (15.30)	26.40 (10.39)	31.35 (14.59)	19.04 (7.49)
pH	6.77 (0.46)	6.98 (0.38)	7.04 (0.42)	6.61 (0.42)	6.65 (0.35)	6.49 (0.44)	6.56 (0.39)	6.59 (0.52)	6.59 (0.34)	6.43 (0.46)	6.60 (0.42)	6.67 (0.31)

Units in mmol∙kg^−1^ (except pH). ∗ = significantly different to week 1 within condition (*p* ≤ 0.02). ^#^ = significantly different to MPI at corresponding visit (*p* = 0.01).

**Table 7:** Summary fermentation metabolites (expressed as sum [Σ], relative percentage [%] and ratio) for each product intervention.

	MPI			HYB			PB			PBF		
	Baseline	Week1	Week2	Baseline	Week1	Week2	Baseline	Week1	Week2	Baseline	Week1	Week2
Σ SCFAs	61.55 (20.85)	60.57 (30.53)	54.20^$^ (25.80)	59.48 (25.82)	51.28^#^ (22.61)	71.94∗ (35.96)	63.02 (26.78)	70.55^#^ (46.12)	77.79^$^ (32.37)	67.52 (27.79)	60.87 (30.46)	60.79 (28.12)
Σ BCFAs	3.90 (1.34)	4.71 (2.60)	4.18 (2.15)	4.25 (3.32)	3.72 (2.24)	3.86 (1.64)	4.70 (2.71)	5.22 (3.43)	5.48 (3.06)	3.97 (1.71)	4.96 (2.48)	3.53 (1.82)
Acetic acid (%)	63.18 (5.39)	61.78 (7.99)	61.17 (6.50)	61.60 (4.82)	62.01 (7.37)	60.80 (5.76)	60.93 (8.35)	61.07 (6.50)	61.62 (6.75)	63.57 (4.95)	60.21 (3.97)	62.10 (4.71)
Propionic acid (%)	18.61 (4.51)	19.79 (3.97)	21.17 (4.39)	19.24 (3.59)	18.75 (5.69)	19.15 (3.91)	19.77 (4.47)	19.62 (3.60)	19.33 (2.53)	17.84 (4.05)	19.11 (3.49)	19.76 (3.06)
Butyric acid (%)	15.90 (3.50)	15.49 (4.74)	15.00 (5.64)	16.38 (4.43)	16.21 (4.47)	17.21 (4.80)	16.42 (5.31)	16.47 (3.59)	16.46 (5.32)	16.37 (4.57)	17.88 (3.69)	15.60 (3.06)
Valeric acid (%)	2.32 (1.04)	2.94 (1.02)	2.67 (0.98)	2.78 (1.19)	3.03 (0.81)	2.84 (1.08)	2.88 (1.64)	2.84 (1.17)	2.58 (1.05)	2.23 (1.28)	2.79 (0.97)	2.53 (0.81)
Σ SCFAs/Σ BCFAs + ammonia	2.75 (1.49)	2.25 (1.54)	1.97 (0.79)	2.30 (1.37)	1.82 (1.25)	3.22∗ (2.78)	2.06 (0.89)	2.11 (1.19)	2.61 (1.19)	2.29 (1.25)	1.76 (0.95)	3.28∗ (2.23)

Units for all metabolites in mmol∙kg^−1^; ∗ denotes difference to week 1 within intervention only (*p* ≤ 0.013). ^#^ denotes difference between products at week 1 (*p* = 0.046). ^$^ denotes difference between products at week 2 (*p* = 0.007).

### 3.5 Bristol stool test evaluation and gastrointestinal symptoms

BST scores indicated normal stool transit throughout, with no significant between-product differences reported (*p* > 0.05; Supplementary Table 2). Minimal gastrointestinal symptoms were reported and generally comparable between interventions (Supplementary Fig. 1); however, an overall difference between MPI and PB for dry mouth was observed at week 2 (*p* = 0.025), with 10 participants reporting mild to moderate responses prior to the week 2 laboratory trial for PB (compared to 3 for MPI), despite following standardised hydration recommendations.

### 3.6 Microbiota assessment

**Fig. 3:**
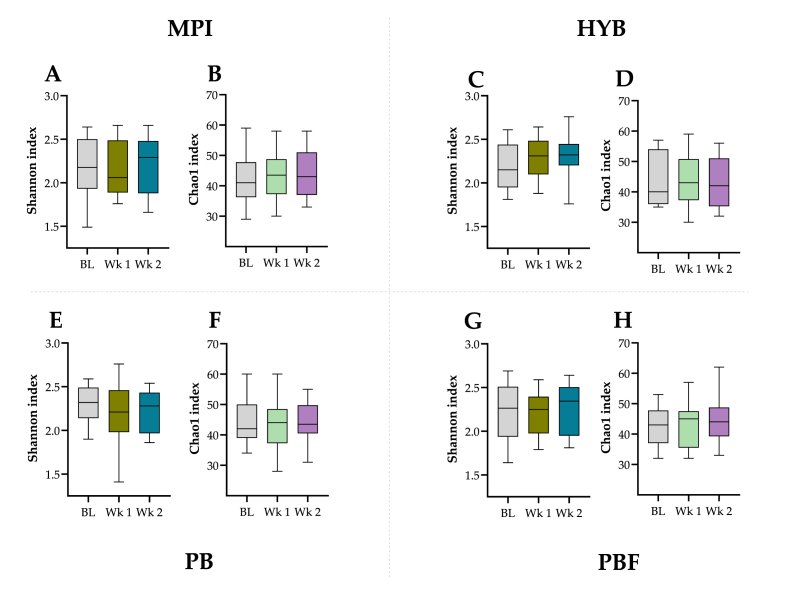
Alpha diversity assessed via Shannon [A, C, E, G] and Chao1 [B, D, F, H] indices for MPI, HYB, PB or PBF at baseline (BL), week 1 (Wk 1) and 2 (Wk 2).

*Alpha diversity:* Alpha diversity was assessed with the Shannon (Fig. 3; A, C, E, G) and Chao1 (Fig. 3; B, D, F, H) indices for all products at baseline, week 1 and week 2. No significant differences were observed in the Shannon index within or between products over time (*p* > 0.05). However, a trend towards an increased Shannon index for HYB from baseline to week 2 (*p* = 0.058) was observed. No significant differences were observed in the Chao1 index within or between products over time (*p* > 0.05).

**Fig. 4:**
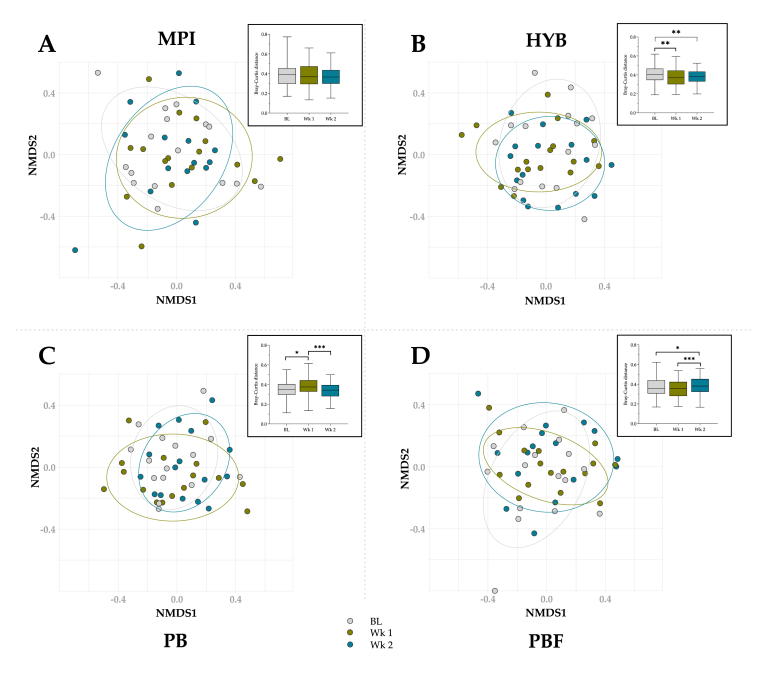
Beta diversity assessed via non-metric multidimensional scaling (NMDS) (main graphs) and Bray-Curtis distance (inserts) for MPI, HYB, PB or PBF at baseline (BL), week 1 (Wk 1) and 2 (Wk 2).∗*p* adjusted ≤0.027 between timepoints; ∗∗*p* adjusted ≤0.007 between timepoints; ∗∗∗*p* adjusted <0.0001 between timepoints.

*Beta-diversity:* Beta-diversity was analysed using non-metric multidimensional scaling (NMDS) (Fig. 4 main graphs) based on Bray-Curtis distances (Fig. 4 inserts). No significant differences were observed within the MPI intervention between timepoints (*p* > 0.05; Fig. 4A insert). Bray-Curtis distances showed significantly lower within intervention variability for HYB at both week 1 (*p* adjusted = 0.007) and week 2 (*p* adjusted = 0.002) compared to baseline (Fig. 4B insert). For PB, within intervention variability increased significantly from baseline to week 1 (*p* adjusted = 0.012) and significantly decreased from week 1 to week 2 (*p* adjusted <0.0001) (Fig. 4C insert). Finally, for PBF, within group variability was significantly increased from baseline to week 2 (*p* adjusted = 0.027) and from week 1 to week 2 (*p* adjusted <0.0001) (Fig. 4D insert). No significant differences were observed between interventions.

**Fig. 5:**
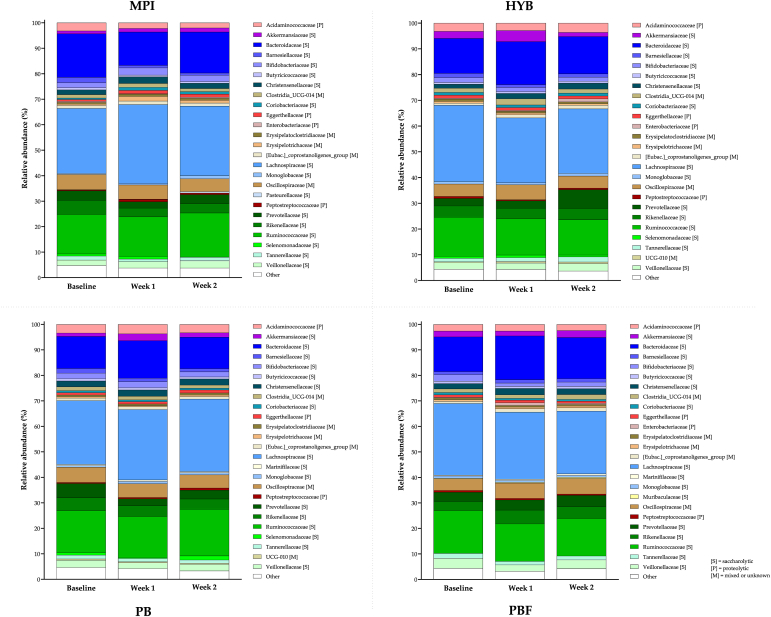
Microbiota composition highlighting the 25 most abundant taxa (family) for MPI, HYB, PB or PBF at baseline, week 1 and 2.

*Microbiota abundance*. Microbiota abundance of the top 25 taxa on family level is shown in Fig. 5. The most abundant taxa in all four products at all three timepoints was the Lachnospiraceae family, followed by the Bacteroidaceae and Ruminococcaceae families in different orders of abundance (second or third most abundant) between the products. Bacteroidaceae were the second most abundant family in MPI at baseline, HYB at week 1 and week 2, PBF at week 1 and 2, followed by Ruminococcaceae. For the other products and weeks Ruminococcaceae were the second most abundant taxa and Bacteroidaceae the third most abundant taxa. These three families contain predominantly saccharolytic bacteria and cover >50% of the microbiota abundance of all four products at all time points (overall mean: 57.4%). Oscillospiraceae, Prevotellaceae and Rikenellaceae families comprised the next most abundant taxa for all products accounting for >11% of the microbiota abundance across all time points (overall mean: 13.7%).

**Fig. 6:**
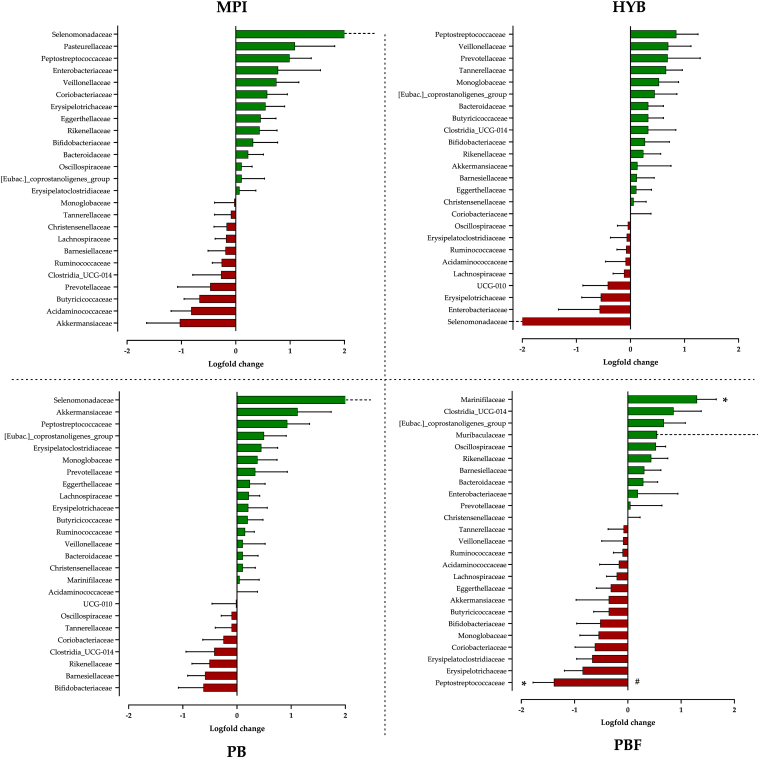
Overall logfold change data for the 25 most abundant taxa (family) for MPI, HYB, PB or PBF. ∗ denotes significant difference within product (*p* ≤ 0.014); ^#^ denotes differences to all other products (*p* ≤ 0.007). Dashed line represents outside range.

Changes in microbiota abundance were assessed on a family level using DESeq logfold changes (Fig. 6). No significant differences were reported for MPI, HYB or PB (*p* > 0.05). However, for PBF, the (saccharolytic) family Marinifilaceae was significantly increased (*p* adjusted = 0.014) within intervention only. In contrast, within the PBF intervention a significant reduction in the (proteolytic) family Peptostreptococcaceae was observed by week 2 (*p* adjusted = 0.014), and in comparison to all other products (*p* adjusted ≤0.0068). As logfold changes >1 are considered clinically meaningful (and >0.5 highlighting smaller taxonomic shifts), it is noteworthy that the Pasteurellaceae family was increased, whereas Akkermansiaceae decreased, for MPI. However, for PB, Akkermansiaceae increased >1 (logfold change; *p* > 0.05). Larger logfold changes (>2) in the Selenomonadaceae family observed for MPI, HYB and PB were based on single participants, likely explaining non-significance. No other differences between or within interventions were observed.

**Fig. 7:**
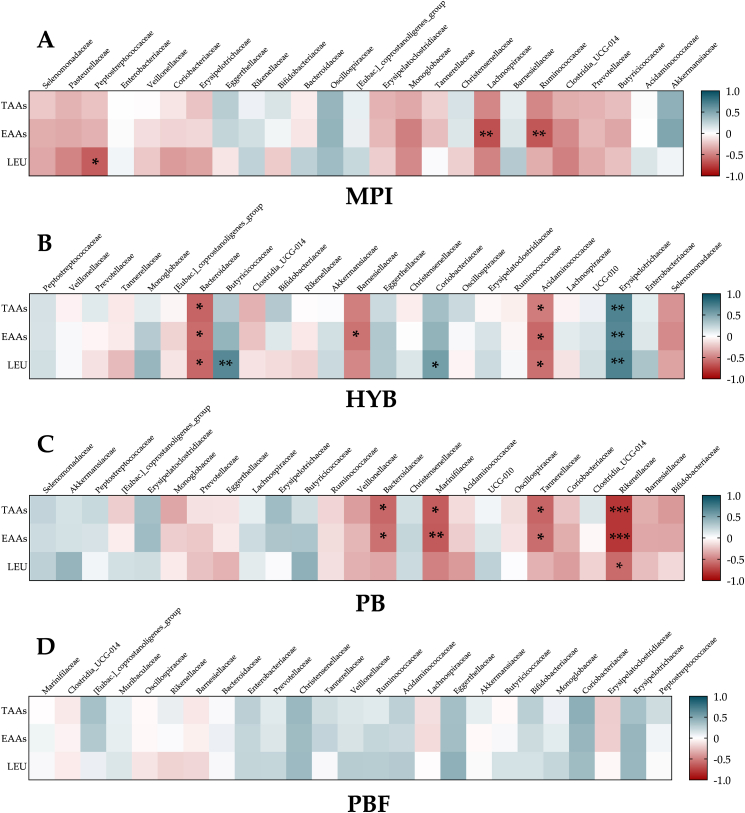
Heatmap correlations (logfold change) between the top 25 taxa specific to each product and amino acids (total [TAAs], essential [EAAs] and leucine [LEU]). ∗ denotes significant correlation (*p* ≤ 0.05); ∗∗ denotes significant correlation (*p* ≤ 0.008); ∗∗∗ denotes significant correlation (*p* ≤ 0.0005). Note: a positive correlation indicates that a reduction in the taxa correlated with decreased bioavailability.

*Heatmap correlations:* When assessing potential relationships between microbiota and amino acid changes (Fig. 7), a significant negative correlation was observed between reduced EAA bioavailability and Lachnospiraceae (r = −0.69) and Ruminococcaceae (r = −0.66) taxa prevalence for MPI (*p* ≤ 0.007). An increased abundance of the Peptostreptococcaceae family with MPI was also negatively correlated with L-leucine (r = −0.64; *p* < 0.01). For HYB, notably the Bacteroidaceae (r = −0.61, r = −0.58, r = −0.59) and Acidaminococcaceae (r = −0.51, r = −0.59, r = −0.56) families were negatively correlated with TAAs, EAAs and L-leucine respectively (*p* ≤ 0.05). However, the Erysipelotrichaceae family demonstrated a positive association with all blood amino acid measures (r = 0.70, r = 0.67, r = 0.70 for TAAs, EAAs and L-leucine respectively; *p* ≤ 0.006) indicating a reduction in this taxon was potentially associated with reduced bioavailability. For PB, a strong negative correlation was observed between the Rikenellaceae family and both TAAs and EAAs (r = −0.79; *p* < 0.001 for both), as well as L-leucine (r = −0.58; *p* = 0.02). Additionally, Bacteroidaceae, Marinifilaceae and Tannerellaceae taxa were also negatively associated with reduced profiles for TAAs and EAAs (r ≥ −0.56; *p* ≤ 0.03). No significant correlations were observed for PBF (*p* > 0.05).

## 4 Discussion

The aim of this study was to assess the acute and sustained effects of different protein sources on amino acid profiles, uremic toxins, faecal fermentation metabolites and microbiota composition in healthy volunteers. At baseline, both PB products resulted in lower TAA profiles compared to MPI and HYB as expected. However, after the 2-week intervention, TAA profiles were not improved with PB or PBF consumption. Furthermore, whilst consumption of products containing casein and whey resulted in a faster uptake of total amino acids after the intervention, TAA profiles only remained similar to baseline with HYB, indicating that a combination of protein sources may be beneficial for both non-essential and essential amino acid supply (Dijk et al., 2023).

A similar pattern was also observed when profiles for all products were compared for EAAs, with findings comparable to other studies (Rogers et al., 2024). EAA profiles did not improve with either PB or PBF following consumption of a higher protein intake from these products (and in fact decreased further after the intervention compared to baseline). Furthermore, at baseline and after the intervention, both PB and PBF resulted in a lower iAUC compared with both MPI and HYB. In contrast, the inclusion of diary proteins within the product (both MPI and HYB) resulted in a faster uptake of essential amino acids at 15 min post intervention. This may be an important factor to consider where ‘fast uptake’ of essential amino acids may be of clinical relevance (Weijs et al., 2014). Whilst increased splanchnic extraction likely explains the relative decrease in EAA profiles and lower iAUC for both PB products, particularly to an increased protein intake (Hojfeldt et al., 2020), it is also viable that more of the PB amino acids and peptides, along with phytonutrients become available in the large intestine for fermentation. Of course, systemic turnover of amino acids and tissue extraction (e.g. muscle) should also be considered when interpreting results (Hojfeldt et al., 2020).

An important finding from the study was that plasma L-leucine profiles were significantly lower with all products after 2 weeks sustained consumption, except for MPI. Furthermore, whilst iAUC for L-leucine was only maintained with MPI, both MPI and HYB resulted in a greater L-leucine absorption at 15 min into the profile assessment at week 2. As L-leucine profiles were reduced at week 2 with PB products, this supports the reasoning that dairy proteins would be more advantageous in the rapid supply of specific amino acids. As L-leucine is an important amino acid in the activation of mammalian target of rapamycin (mToR) (Koopman et al., 2006), optimal supply of dairy (i.e. whey) constituents would be deemed relevant when muscle protein synthesis (MPS) is considered (Pennings et al., 2011). Whilst MPS rates were not assessed in the current study, previous research has indicated that habituation to higher protein intakes does not necessarily offer substantial improvements in younger (Hursel et al., 2015) or older healthy adults (Gorissen et al., 2017). However, recent research has also indicated that, if provided at sufficiently high levels, the type of proteins used in PB blends may also demonstrate equivalence to dairy protein in terms of myofibrillar protein synthesis rates (Van Der Heijden et al., 2024). Therefore, with the reduced EAA and L-leucine profiles observed with PB blends, further research is required to assess whether the pea and soy isolates employed in the current study may also be beneficial in terms of MPS, at what specific dose, and particularly if fortified with L-leucine (Lim et al., 2024). It is also important to note that whilst protein quantity of products was determined according to manufacturer certification, independent analysis of individual amino acids indicated that total and essential amino acids were greater with both MPI and HYB, likely contributing to these findings. However, analysis of PB products were in line with expected quantities supporting the within-product finding that amino acid profiles were reduced following sustained consumption of both PB products.

Changes in uremic markers were largely unremarkable, indicating that an elevated intake of total protein did not impact the production of uremic toxins dramatically in healthy volunteers. This is a useful finding in that dietary intake of protein increased from ∼1.3 g· kg^−1^ to 2.0 g ·kg^−1^ with the inclusion of daily protein supplementation. In healthy volunteers, this would be considered a high protein intake, and hence, with increased proteolytic fermentation, an increase in such markers would potentially be expected (Rodriguez-Romero et al., 2022). Overall amino acid profiles, particularly EAAs, were reduced by week 2, indicating that a greater proportion of amino acids may have been taken up or metabolised by splanchnic tissues and/or reach the colon. Only indoxyl sulfate (IS) concentration was increased within intervention for each of the PB related products. This suggests that the inclusion of plant-based proteins may have impacted bacterial taxa pertinent to tryptophan metabolism to indole (Patel et al., 2012). In the present study, involving healthy individuals, the concentration of IS was considered normal (Duranton et al., 2012; Vanholder et al., 2003). As such, whilst considered a uremic toxin (Leong and Sirich, 2016), a small increase in IS may have beneficial implications for gut mucosal tight-junction integrity (Candeliere et al., 2022; Vanholder et al., 2022), through activation of the aryl hydrocarbon receptor (AhR) and interleukin-22 production (Rodriguez-Romero et al., 2022). For PBF it was also noted that a small increase in p-cresyl glucuronide (p-CG) was observed by week 2. Modulation of specific bacterial taxa (e.g. Clostrodia, which subtly shifted with PBF; logfold change: 0.9 ± 0.5) may have led to more selective metabolism of aromatic amino acids (particularly L-tyrosine) and may provide substrates (glucuronic acid) for microbiota metabolism (Blachier and Andriamihaja, 2022). However, with the hypothesis of an increase in saccharolytic fermentation with PBF, it was expected that any changes in uremic markers would be negative, particularly with additional fibre (Patel et al., 2012; Sinha et al., 2024). At low relative physiological concentrations, small increases in selected uremic markers, such as p-CG and IS, may therefore be specific to PB or fibre inclusion reflecting acute microbiota metabolism.

It was theorised that PB and the inclusion of fibre within the supplemental interventions would support an increase in saccharolytic fermentation, in essence counteracting expected proteolytic fermentation with higher protein intakes. For fermentation metabolites, minimal effects were observed for single analytes, despite within-group effects for HYB for increased acetic and butyric acid. Additionally, despite a notable decrease in ammonia by week 2 for PBF, this reduction was not significant. However, when variables were collated, an increase in total SCFAs was observed for HYB, and particularly PB, over the intervention. Surprisingly, the addition of fibre did not impact total SCFA production. One explanation for this may have been the habitual dietary fibre intake in such healthy individuals. To provide a proxy evaluation of saccharolytic: proteolytic fermentation, we assessed the ratio between ΣSCFAs and ΣBCFAs + ammonia. Whilst no between-intervention differences were observed, it was noted that both HYB and PBF increased this ratio by week 2 within-intervention. This supported, in part, our theory highlighting the acute interaction between short- and branched-chain fatty acids/proteolytic metabolites and suggests that the combination of ingredients (pea/soy with dairy proteins in HYB, or the additional mixed fibres in PBF) may have led to competitive production of SCFAs or reduction in BCFAs/ammonia, ultimately resulting in a switch towards saccharolytic fermentation (Jardon et al., 2022). This may have important implications for host physiology and requires further investigation particularly in clinical populations or longer-term high protein intakes (Cai et al., 2022).

In terms of microbiota adaptations, whilst alpha diversity was largely unaffected, within intervention changes were noted for beta diversity, in particular an increase by week 2 with PBF which was not observed in the other interventions. As such, the inclusion of a mixed fibre may have supported greater taxonomic divergence. Aligned with this, a key finding from this study was that PBF resulted in a significant and meaningful reduction in the Peptostreptoccaceae taxa compared to all interventions. Additionally, PBF resulted in a significant increase in the Marinifilacaeae taxa across the intervention. Whilst assessment of individual taxa should not be taken out of context, this does suggest that inclusion of the fibre in PBF resulted in competitive growth potentially favouring saccharolytic fermentation (Partanen et al., 2024). Peptostreptoccaceae have been associated with proteolytic fermentation along with more opportunistic pathogen development (Zaccaria et al., 2023). In contrast, Marinifilacaeae members, for instance *Butyricimonas* species (Lee et al., 2022; Takasugi et al., 2025), have been associated with mucosal integrity and reduced systemic inflammation potentially related to their butyrate production capacity (Singh et al., 2022), thereby acting in a protective manner. However, whilst not statistically significant, it is also noteworthy that for both PB products, a reduction was observed (based on logfold changes) in otherwise beneficial taxa including Bifidobacteriaceae, Coriobacteriaceae and Tannerellaceae. As such, each protein intervention resulted in minor, but unique microbiota ‘signatures’ based on the most abundant taxa and the type of protein consumed. Whilst all attempts were made for participants to maintain normal habitual dietary patterns, it should also be noted that dietary variance over each intervention likely contributed to these findings. However, the inclusion of a mixed fibre complex with pea/soy protein supplementation resulted in potentially beneficial taxa shifts which requires further investigation.

Finally, although it is difficult to determine causality between taxonomic changes and amino acid bioavailability, several taxa were highlighted as significantly correlated to reduced total and essential amino acid profiles. In particular, for HYB, a reduction in Erysipelotrichaceae, whereas subtle changes in Bacteroidacceae and Acidaminococcaeae, may be associated with observed reductions in amino acid profiles. For PB, the Rikenellaceae taxa were also associated with reduced amino acid profiles. However, no associations were found for PBF. These findings are unclear but may infer that when mixed protein strategies are employed, competitive and selective changes in specific taxa may modulate amino acid metabolism directly or via transporter expression (Han et al., 2025). However, these findings should be interpreted with caution.

It is important to note several limitations to the current study. Firstly, whilst participants were requested to consume typical dietary intakes, there were inter-individual variances observed. Whilst mean protein intake was considered moderate at ∼1.3 g·kg^−1^, intake ranged from 0.7 to 2.2 g·kg^−1^ reflecting typical patterns observed between otherwise healthy individuals. Likewise, whilst fibre intake was considered satisfactory at ∼25 ± 11 g·d^−1^, variability between individuals and potentially across the supplemental intervention may have impacted findings. It should also be considered that fortification with fibre added to PBF may have been negated by the dietary intake which in this population almost met the daily recommendations. Additionally, inherent fibre present in plant-based sources (particularly PB) may have limited findings.

Although a strength of the study was the employment of randomised, controlled and cross-over conditions, recruitment of a mixed cohort may also limit sex-specific interpretations. Future research should consider whether increased (and longer term) protein intake impacts amino acid profiles and/or taxonomic changes in a sex-specific manner and across larger populations. A further limitation of the study was that protein content was not ascertained in faecal samples. This may have been beneficial to determine whether increased quantities of dietary protein were in fact reaching the large intestine, supporting within-intervention findings specific to saccharolytic: proteolytic fermentation. Finally, it would have been beneficial to have undertaken metagenomic evaluation of samples which may provide more functional or translational insights of relatively small changes in microbiota taxa.

## 5 Conclusion

Plant-based protein strategies, when consumed regularly (50 g·d^−1^) as part of an otherwise mixed habitual diet, resulted in reduced amino acid profiles both acutely and following a two-week intervention in comparison to dairy proteins. As such, sustained intake of both PB or PBF did not result in improved amino acid profiles, particularly relevant to the provision of essential amino acids after each intervention. Only milk protein isolates maintained relative bioavailability of L-leucine, which may have important clinical applications or when considering muscle protein synthesis. Changes in taxonomic microbiota were unique to each intervention, and reflective of minor adaptations. However, when coupled with findings from fermentation metabolites, the inclusion of a mixed fibre blend to a plant-based strategy may impact specific taxa pertinent to saccharolytic fermentation, which may have important protective health benefits. Further research is warranted to confirm these findings in both healthy and clinical populations.

## Ethical approval and consent to participate

This study was undertaken following institutional ethical approval from the Faculty of Science and Engineering Research Ethics Panel, Anglia Ruskin University (Ethical approval no. ETH2223-3364) and was registered with ClinicalTrials.gov (ID: NCT05669612). All laboratory-based procedures were conducted in accordance with the Declaration of Helsinki (World Medical Association, 2025) at the Human Physiology Laboratory, Anglia Ruskin University, Cambridge, UK. All participants provided written, informed consent prior to study inclusion.

## Publication statement

This manuscript has not been published elsewhere and has not been submitted simultaneously for publication elsewhere.

## CRediT authorship contribution statement

Justin Roberts: Conceptualisation, Project administration, Methodology, Investigation/Data Collection, Formal Analysis, Writing – original draft, Writing – review and editing. Joseph Lillis: Project administration, Investigation/Data Collection, Formal Analysis, Writing – original draft, Writing – review and editing. Jeff Mercer: Investigation/Data Collection, Writing – review and editing. Helen Mercer: Investigation/Data Collection, Writing – review and editing. Ioannis Kostopoulos: Investigation, Formal Analysis, Writing – original draft, Writing – review and editing. Lotte Dopheide: Investigation, Formal Analysis, Writing – original draft, Writing – review and editing. Ashley Willmott: Investigation/Data Collection, Writing – review and editing. Sebastian Tims: Investigation, Formal Analysis, Writing – original draft, Writing – review and editing. Matthew Furber: Conceptualisation, Writing – review and editing. Ardy van Helvoort: Conceptualisation, Interpretation, Writing-review and editing. Jorge Marques Pinto: Project administration, Investigation/Data Collection, Formal Analysis, Writing – original draft, Writing – review and editing.

## Funding

This research was funded by a grant from 10.13039/100007773Danone Research & Innovation, Utrecht, The Netherlands (Project Code: R9097; 10.13039/100010330Anglia Ruskin University, Cambridge, UK). All laboratory trials, participant management and study co-ordination was undertaken independently of the funding company.

## Declaration of competing interest

The authors declare the following financial interests/personal relationships which may be considered as potential competing interests: Justin Roberts reports financial support was provided by Danone Research & Innovation, Utrecht, The Netherlands. Justin Roberts reports a relationship with Danone Research & Innovation, Utrecht, The Netherlands that includes: employment. Ioannis Kostopoulos reports a relationship with Danone Research & Innovation, Utrecht, The Netherlands that includes: employment. Lotte Dopheide reports a relationship with Danone Research & Innovation, Utrecht, The Netherlands that includes: employment. Sebastian Tims reports a relationship with Danone Research & Innovation, Utrecht, The Netherlands that includes: employment. Matthew Furber reports a relationship with Danone Research & Innovation, Utrecht, The Netherlands that includes: employment. Ardy van Helvoort reports a relationship with Danone Research & Innovation, Utrecht, The Netherlands that includes: employment. If there are other authors, they declare that they have no known competing financial interests or personal relationships that could have appeared to influence the work reported in this paper.

## Data Availability

The data presented in this study is available upon request from the corresponding author. The data is not publicly available due to ethical considerations, in accordance with participant consent on the use of confidential data.
