# Supplementary material for: Comparative effects of dairy, hybrid and plant-based protein blends (including fibre fortification) on amino acid profiles and gut microbiota adaptations: The Promephy study
Source: Curr Res Food Sci. 2026 Feb 19;12:101359. doi: 10.1016/j.crfs.2026.101359 (PMC12963923; doi:10.1016/j.crfs.2026.101359)
Supplement: Multimedia component 1 [file mmc1.docx]

Supplementary Figure 1. Mean symptom and gastrointestinal tolerance responses during laboratory trials. Ab.=abdominal. * = overall difference between MPI and PB at week 2 (*p* = 0.025).
