# Supplementary material for: Comparative effects of dairy, hybrid and plant-based protein blends (including fibre fortification) on amino acid profiles and gut microbiota adaptations: The Promephy study
Source: Curr Res Food Sci. 2026 Feb 19;12:101359. doi: 10.1016/j.crfs.2026.101359 (PMC12963923; doi:10.1016/j.crfs.2026.101359)
Supplement: Multimedia component 2 [file mmc2.docx]

Supplementary Table 1. Mean physical activity parameters relative to each product intervention.

|  | **MPI** | **HYB** | **PB** | **PBF** |
| --- | --- | --- | --- | --- |
| Number of sessions | 10 (4) | 11 (6) | 10 (5) | 11 (5) |
| Session duration (mins) | 53 (15) | 56 (15) | 61 (20) | 57 (20) |
| Training load (AU) | 2572 (1671) | 2404 (1409) | 2752 (1846) | 3566 (3502) |
| Heart rate (b·min^-1^) | 124 (24) | 123 (21) | 121 (23) | 122 (21) |
| sRPE | 5 (2) | 4 (1) | 5 (1) | 5 (2) |

AU = arbitrary units; sRPE = Session rating of perceived exertion. No significant differences observed.
