# Supplementary material for: Comparative effects of dairy, hybrid and plant-based protein blends (including fibre fortification) on amino acid profiles and gut microbiota adaptations: The Promephy study
Source: Curr Res Food Sci. 2026 Feb 19;12:101359. doi: 10.1016/j.crfs.2026.101359 (PMC12963923; doi:10.1016/j.crfs.2026.101359)
Supplement: Multimedia component 3 [file mmc3.docx]

Supplementary Table 2. Mean Bristol stool test (BST) data for each product intervention, including relative distribution.

|  | **MPI** | | | **HYB** | | | **PB** | | | **PBF** | | |
| --- | --- | --- | --- | --- | --- | --- | --- | --- | --- | --- | --- | --- |
|  | Baseline | Week1 | Week2 | Baseline | Week1 | Week2 | Baseline | Week1 | Week2 | Baseline | Week1 | Week2 |
| BST Score | 3.63 (1.26) | 2.88 (1.41) | 3.19 (1.17) | 3.63 (1.26) | 3.31 (1.30) | 3.75 (1.48) | 3.31 (0.95) | 3.13 (1.20) | 3.44 (1.36) | 3.69 (1.30) | 3.00 (0.73) | 3.19 (1.05) |
| C1 (%) | 25.0 | 43.8 | 37.5 | 18.8 | 31.3 | 25.0 | 12.5 | 31.3 | 25.0 | 25.0 | 25.0 | 25.0 |
| C2 (%) | 62.5 | 50.0 | 56.3 | 75 | 62.5 | 56.2 | 87.5 | 62.5 | 62.5 | 62.5 | 75.0 | 68.8 |
| C3 (%) | 12.5 | 6.2 | 6.2 | 6.2 | 6.2 | 18.8 | 0.0 | 6.2 | 12.5 | 12.5 | 0.0 | 6.2 |

C1 = category 1, mean of BST 1-2 scores (representative of ‘*slow transit*’); C2 = category 2, mean of BST 3-5 scores (representative of ‘*normal transit*’); C3 = category 3, mean of BST 6-7 scores (representative of ‘*fast transit*’). No significant differences found within or between products.
